# Duration and quality of the peer review process: the author’s perspective

**DOI:** 10.1007/s11192-017-2310-5

**Published:** 2017-03-09

**Authors:** Janine Huisman, Jeroen Smits

**Affiliations:** 1 Department of Business Administration, Institute for Management Research, Radboud University, Nijmegen, The Netherlands; 2 SciRev Foundation, Nijmegen, The Netherlands; 3 Department of Economics, Institute for Management Research, Radboud University, Nijmegen, The Netherlands

**Keywords:** Peer review process, Duration, Quality, Author’s experience

## Abstract

To gain insight into the duration and quality of the scientific peer review process, we analyzed data from 3500 review experiences submitted by authors to the SciRev.sc website. Aspects studied are duration of the first review round, total review duration, immediate rejection time, the number, quality, and difficulty of referee reports, the time it takes authors to revise and resubmit their manuscript, and overall quality of the experience. We find clear differences in these aspects between scientific fields, with Medicine, Public health, and Natural sciences showing the shortest durations and Mathematics and Computer sciences, Social sciences, Economics and Business, and Humanities the longest. One-third of journals take more than 2 weeks for an immediate (desk) rejection and one sixth even more than 4 weeks. This suggests that besides the time reviewers take, inefficient editorial processes also play an important role. As might be expected, shorter peer review processes and those of accepted papers are rated more positively by authors. More surprising is that peer review processes in the fields linked to long processes are rated highest and those in the fields linked to short processes lowest. Hence authors’ satisfaction is apparently influenced by their expectations regarding what is common in their field. Qualitative information provided by the authors indicates that editors can enhance author satisfaction by taking an independent position vis-à-vis reviewers and by communicating well with authors.

## Introduction

The scientific peer review process is one of the weakest links in the process of scientific knowledge production. While it is possible to review a paper in less than a day (Ware and Mabe 2015), it may often lie untouched on reviewers’ desks and in editorial offices for extended periods before it is evaluated. This means a substantial loss of time for the scientific process, which has otherwise become much more efficient in the last decades. There are even indications that the duration of the peer review process may have increased in the last decades (Ellison 2002a; Azar 2007). Hence there are good reasons for a critical look at this process.

To gain insight into the duration and other key aspects of the peer review process, we analyze data from 3500 review experiences submitted by authors to the SciRev.sc website (www.scirev.sc). On this website, researchers can share their experiences with the peer review process regarding manuscripts they have submitted to scientific journals. This information can subsequently be used by their colleagues when selecting a journal to submit their work. Information is available on several important aspects of the peer review process, including the duration of the first review round, total review duration, the time editors take to inform authors about an immediate (desk) rejection of a manuscript, the number and quality of referee reports, the time authors take to revise and resubmit their manuscript, and the overall quality of the process as experienced by the authors.

Duration of the first review round—or first response time (Azar 2007)—is probably most important for scientific authors as it determines how much time may be lost if the outcome is negative (Solomon and Björk 2012). The number of review rounds and the time journals take to manage these rounds are also important, as these aspects significantly affect the time that elapses until author(s) are informed of the final editorial decision. Another important duration indicator is the immediate (desk) rejection time, i.e., the time taken by an editor to inform authors that the manuscript is not considered fitted for the journal. If this only takes a few days, authors can without much time loss send the manuscript to another journal. However, quite often, editors may take weeks or even months for a desk rejection. This seems unacceptable and may point to a less than efficient organization of the editorial process. If editors take much time to inform authors that they are not interested in the manuscript, they probably will also be rather slow in other aspects of manuscript handling, such as assigning reviewers and processing review reports. The immediate rejection time is thus a major indicator of a journal’s performance.

Besides by the duration of the different steps of the peer review process, total publication time is also influenced by revision time, i.e., the time taken by authors to revise and resubmit the manuscript. This factor is therefore also included in our analysis. It is influenced by the time authors are able and prepared to spend on the revision of the manuscript and by the difficulty of the revisions required. In this connection, it is important also to include aspects of the referee reports. Constructive comments by reviewers may substantially contribute to the quality of scientific papers, while low quality and contradictory referee reports may be a major source of frustration among authors (Nicholas et al. 2015). In the SciRev questionnaire, authors are asked about the number of reports they received and how they experienced the quality of the reports and the difficulty of the changes they were required to make.

Besides the measurable factors, such as the duration of the different phases of the peer review process and the number of referee reports, there are also aspects of the process that are more difficult to quantify. Does the editor take questions of the author(s) seriously? Is a reasonable motivation for a (desk) rejection given? Does the editor take an independent position vis-à-vis reviewers when making important decisions? Does the editor advise authors on the importance of specific reviewer comments? Together these aspects affect the author’s experience with the journal and to a certain extent may turn a rejection into a good experience or an acceptance into a bad one. We therefore also analyze the authors’ overall evaluation scores given to the journals for their peer review performance as well as the motivations given by authors for their scores. Because an author’s review experience is influenced by many factors (e.g., the outcome of the review process, the impact factor of the journal, and differences in expectations between scientific fields), we study the overall scores in a multivariate way and also analyze the authors’ scoring motivations.

## Background

There are around 28,000 scientific journals worldwide, which publish 2.5 million scientific articles annually, produced by a research community of 6–9 million scientists (Ware and Mabe 2015; Jinha 2010; Björk et al. 2009; Plume and Van Weijen 2014; Etkin 2014). Many of the published articles have been rejected at least once before they reached the editor’s desk of the journal in which they were published. This means that each year many more manuscripts pass through peer review than are published.

Although there is some variation among journals, the peer review process typically starts with a first evaluation of the manuscript by the editor, followed by a decision to accept the manuscript for peer review or immediately (desk) reject it. If desk rejected, the corresponding author receives a message from the editor that the manuscript is considered not fit for publication in the journal, with or without a brief motivation given for the rejection. A manuscript that has passed this first stage will then be send out for peer review, whereby experts in the field (peers of the authors) evaluate the manuscript and write a referee report. On the basis of these reports, the editor decides either to reject the manuscript or gives the author(s) an opportunity to revise and resubmit it, or—in exceptional cases,—directly accepts it. In case of a revise-and-resubmit, several additional review rounds may follow before a final decision regarding acceptance or rejection is made. If the process takes exceptionally long, the author may decide to withdraw the manuscript and submit it to another journal.

### Process too slow

Given the fact that reviewers are often overloaded with academic work, that they are generally not paid for their review work, and that reviews are mostly anonymous, there are few incentives to give high priority to this work (Azar 2007; Moizer 2009). Hence, while the actual time it takes to write a referee report may vary between a few hours and a day (Ware and Mabe 2015), reviewers tend to take several weeks to several months to submit their reports. Apart from the time reviewers take to deliver their reports, the total manuscript processing time of journals is influenced by the duration of the various stages of manuscript handling at editorial offices. Given that these offices often have limited resources and many editors do this work besides busy academic careers, waiting times at the different stages are often (much) longer than strictly necessary.

It is therefore not surprising that one of the most important criticisms of the peer review system is that it is much too slow (Lotriet 2012). There are even indications that is has been getting slower in recent decades (Alberts et al. 2008). Ellison (2002a, 2002b) documents a slowdown since the 1970s in submission-acceptance duration in economics and suggests a similar slowdown in other fields. A major cause for this is that authors are required to revise their manuscripts more often and more extensively (Ellison 2002a, 2002b; Azar 2007; Cherkashin et al. 2009; Björk and Solomon 2013). According to Ellison (2002a), review rounds are of quite recent date. In the early 1950s, ‘almost all submissions were either accepted or rejected: the noncommittal “revise-and-resubmit” was reserved for exceptional cases (p. 948).’

From the author’s perspective, first response time is particularly important, i.e., the time that elapses between submission and first response from the editor, be it rejection, acceptance, or a revise-and-resubmit. First response time is important because it often delays the publication of an article more than once, as many manuscripts are rejected once or several times before acceptance (Azar 2007; Etkin 2014; Pautasso and Schäfer 2010). There are indications that duration of the first review round has increased, at least in some fields. Azar (2007) finds that first response time for economic journals “grew from about 2 months circa 1960 to about 3–6 months in the early 2000s (Azar 2007, p. 182)”. However, as Azar points out, a longer first response time is in itself not necessarily negative. Economics manuscripts have become longer over time and have more mathematical content, which means it is more time-consuming to evaluate them.

### Field difference

Durations vary substantially between scientific fields and even within the same broader discipline. Kareiva et al. (2002), for instance, studying conservation biology, found that the process from submission to publication took on average 572 days for conservation and applied ecology journals compared to 249 days for genetics and evolution journals.

With respect to the number of times the average manuscript is rejected before it reaches the journal that will publish it, Azar (2004) arrives at a figure of three to six rejections. Similar to an increase in first response time, there also seems to be an increase in the number of rejections prior to publication. Thomson Reuters (in Ware and Mabe 2015, p. 51) reports an increase in the rejection rate from 59 to 63% between 2005 and 2010. Regarding the desk rejection rate, Lewin (2014) reports an increase of up to three times for some journals. Lewin attributes this to increased publication pressure, whereby “governments in countries outside of the USA engage in a process of quantifying the scholarship of scientists in their countries as a way of rationalizing the allocation of national resources to institutions of higher learning in their countries. The unsurprising consequence has been a dramatic increase in submissions to the top journals by scholars from emerging economies as well as from European countries” (Lewin 2014, p. 169).

Editors are also worried about these developments. ‘Amongst journal editors there are growing concerns that the quality—and duration—of the review process is being negatively affected as “referees are stretched thin by other professional commitments”. This often leads to “challenges in finding sufficient numbers of reviewers in a timely manner” (Lotriet 2012, p. 27).’ Once reviewers have been found, other problems may emerge, such as poor reviewer agreement on submissions (Peters and Ceci 1982; Onitilo et al. 2014) or ethical problems (Resnik et al. 2008). Reviewers who make contradictory comments are a major source of frustration for authors as well as editors. Regarding unethical practices, Resnik et al. (2008) mention (in order of frequency) reviewers asking authors to include ‘unnecessary references to their publication(s), personal attacks, reviewers delaying publication to publish a paper on the same topic, breach of confidentiality and using ideas, data, or methods without permission (p. 305)’.

### Ways to improve

Several suggestions have been done to make it more attractive for scientists to act as reviewers. Free subscription to journal content, annual acknowledgement on the journal’s website, more feedback about the outcome of the submission and quality of the review, appointment of reviewers to the journal’s editorial board and financial incentives (Tite and Schroter 2007). A noteworthy initiative in this respect is Publons (www.publons.com), a website where reviewers can upload information on anonymous review work they performed. This information is then verified with the journals and can subsequently be used as ‘proof’ of the peer review work done by the reviewer. This initiative provides a solution to the recognition problem. However, it does not help solve the problems of duration and quality as neither the time reviewers spent writing the reports nor the quality of their reports are registered.

As to financial incentives, Thompson et al. (2010) found a statistically significant reduction in review duration when referees were paid for their efforts. ‘Median first response time was reduced from 90 to 70 days, a 22% reduction in the presence of payments. With payments, only 1% of first response times exceeded 6 months; without payments, 16% exceeded 6 months (Thompson et al. 2010, p. 678).’ Although it was not possible to compare the quality of referee reports submitted with or without payment, they thought it likely that if the length of referee reports was an indication of quality, payment might even have led to an increase in referee reports’ quality: “[r]eferees did not dash off shorter reports to meet the deadline for payment; in fact, reports were statistically significantly longer with payments than they were prior to payments” (Thompson et al. 2010, p. 690).

Previous studies by Hamermesh (1994) for seven journals in 1989 also found an increase in timely referee reports for journals offering payments. However, since “some empirical evidence suggests that when voluntary economic activities—giving blood, volunteering to work for public or private institutions, and collecting donations for charity, for example—are rewarded with relatively low payment levels, low-paid performance is inferior to voluntary performance” (Thompson et al. 2010, p. 680), most likely reviewers would have to receive a realistic rather than a symbolic payment for their efforts.

It seems natural to expect that authors of papers that have been accepted are happier with the review experience, when they look back at it in hindsight. Authors tend to suffer from attributional bias. If their paper is rejected, many authors tend to blame this on situational factors, such as incompetent reviewers or uninterested editors, but in case of acceptance tend to attribute this to their own expertise and competence in writing high-quality papers (Garcia et al. 2016). The difference in ratings between authors of accepted and rejected manuscripts might also be greater, the longer the duration of the peer review process. The more time and energy authors invest in a manuscript, the more likely it is they will be disappointed by a rejection, and even more so if rejection follows after several review rounds.

## Methods

The data used in this paper are based on 3500 review experiences, reported by authors between 2013 and 2016, by filling in a questionnaire on the SciRev.sc website. The SciRev questionnaire contains questions on the duration of the different phases of the peer review process of research articles, on the number, quality, and difficulty of the received referee reports, on the outcome of the peer review process, and on whether the manuscript has previously been submitted to another journal. It also asks authors to provide an overall rating of the review experience and gives them the opportunity to motivate their rating. Research articles may include any paper submitted to a scientific journal (regular research papers, review articles, rapid communications, research notes, etc.), provided it has been subjected to peer review.

Authors who submitted a review to SciRev.sc were asked about their affiliation, which was checked by asking them for their institutional email address and sending a confirmation link to that address. Authors who registered with a noninstitutional email address, because for various reasons they could not provide an institutional one (e.g., job change or working in a non-Western institute without good ICT services), were asked for additional information to check their identity. Reviews were only accepted if the author’s identity was confirmed. Reviews of accepted papers were additionally checked at the journals’ websites; these reviews were only included if the author had indeed published a paper in the journal during the period mentioned.

Although the data are not based on a representative sample of author experiences, they are interesting because they paint a broad picture of the range of author experiences from different fields of study. Each submitted review represents the experience of an author and is important as such. If other authors report similar experiences, this would point toward a specific pattern. And if the resultant patterns differ among scientific fields, this would indicate that the prevalence of specific experiences differs among those fields.

There is little reason to expect authors from different fields to be fundamentally different in the way they experience the different aspects of the peer review process. However, there might be different expectations between fields about review duration and hence about what is considered a long process. Besides by field differences, experiences may also be colored by the process outcome and the journal’s impact factor. We therefore split the figures presented in this paper according to scientific field and process outcome (accepted/rejected) and also study relationships with the journal’s impact factor. Information on the impact factor was derived from the journal’s website and other Internet sources. This information could be found for 3126 reviews. In our analysis, we use the natural logarithm of the impact factor, as more journals are concentrated in the lower ranges of the impact factor.

Of the 3500 review experiences, 572 (16.3%) referred to manuscripts that were rejected without being sent to reviewers, 693 (19.8%) that were rejected after the first review round, 2128 (60.8%) that were accepted after one or more review rounds, 43 (1.2%) that were immediately accepted without peer review process, and 64 (1.8%) that were withdrawn by the author. Given the relatively small number of reported cases of manuscripts that were withdrawn or immediately accepted, these were not included in our analysis. We also removed some extreme cases regarding immediate rejection time (>62 days; 53 cases), duration of first review round and total review duration (>100 weeks; 15 cases), and duration of revision after first review round (>300 days; 6 cases). The extreme cases were not concentrated in specific fields.

Information on the various aspects of the peer review process is presented for all review experiences, separately for accepted and rejected papers and for ten major scientific fields: (1) General journals (*n* = 172), (2) Natural sciences (*n* = 1408), (3) Engineering (including technology; *n* = 518), (4) Mathematics and Computer sciences (*n* = 375), (5) Medicine (*n* = 640), (6) Public health (including health professions; *n* = 348), (7) Psychology (including education; *n* = 355), (8) Economics and Business (including law; *n* = 318), (9) Social sciences (*n* = 553), and (10) Humanities (*n* = 178). Given that a substantial number of journals have a broad scope and therefore include more than one scientific field, the sum of the reviews in the different fields is higher than the total number of reviews.

At the end of the SciRev questionnaire, authors are asked to give an overall rating of their review experience. Because this experience is influenced by many aspects of the peer review process, besides providing descriptive figures, also a multivariate regression analysis is performed. In this analysis, the variation in the rating is explained on the basis of relevant characteristics of the process, i.e., whether or not the paper was accepted or rejected, the duration of the first review round, the number of review rounds, the number of referee reports received in the first review round, whether the author is from an English-speaking country, and the scientific field of the journal. We present both direct effects of these factors and significant interactions between them. For journals covering several scientific fields, we only included the journal’s main field in this analysis.

In the multivariate analysis, we excluded reviews of papers that were withdrawn, immediately accepted, or desk rejected. Among the remaining 2821 reviews, there were some missing values. Five reviews for which duration of the first review round was missing were given the average duration of the first review round. Two reviews where the language of the reviewer was missing were included in the non-English (biggest) category. For 289 cases the impact factor was missing. These missings were addressed using the dummy variable adjustment procedure [imputing the mean and including a dummy indicating the missings (cf. Allison 2001)]. Results of the analysis with missing values dealt with in this way were substantially the same as those with all missings removed from the data.

The overall rating of the review experience is measured on a scale running from 0 (very bad) to 5 (excellent). The outcome of the peer review process is a dummy indicating whether the paper was accepted (1) or rejected (0). The duration of the first review round is measured in days. To indicate language background, we included a dummy indicating whether (1) or not (0) the organization where the author works is located in a country where English is the main language used in daily life (i.e., United Kingdom, Ireland, USA, Canada, Australia, New Zealand, South Africa, and British Indian Ocean Territory). Of the 3500 reviews, 2516 were submitted by authors from non-English-speaking countries. Regarding the distribution of reviews over continents, 557 were obtained from Canada and the USA, 96 from Latin America and the Carribean, 2099 from Europe, 470 from Asia and the Pacific, 190 from the Middle East, 83 from Africa, and 5 of which the continent is not known. For the dummies for scientific field, deviation from mean (effects) coding is used. The dummies therefore indicate to what extent the overall rating within the field is higher or lower than the mean of the fields (Hardy 1993).

After rating the overall review experience, authors are given the opportunity to motivate their rating in a few words or sentences. These motivations are published online with the reviews, if permission is given by the author. They paint a sometimes revealing picture of what researchers experience in their attempts to get their work published. To supplement the figures presented in this paper with qualitative information, we analyzed the 1879 motivations available in the 3500 reviews studied.

## Results

### First response time

For authors, the duration of the first review round, or first response time, is probably the factor they are mostly interested in, as this takes up a substantial part of the total manuscript evaluation time and to a large extent determines how much time is lost if the outcome is negative. First response time includes the time taken by the journal for a first evaluation of the manuscript, finding reviewers, the time the latter require to do their work, and the time the editor then requires to evaluate the manuscript in light of the referee reports and to inform authors about the decision.

As can be seen in Table 1, the reported first response time in the SciRev data is on average 13 weeks and varies considerably among scientific fields. It took 8–9 weeks in Medicine and Public health related journals, 11 weeks in Natural sciences and General journals, 14 in Psychology, and 16–18 weeks in Social sciences, Humanities, Mathematics and Computer sciences, and Economics and Business. These figures differ between accepted and rejected manuscripts, with first response time of rejected manuscripts taking, on average, 4 weeks longer.

**Table 1:** First response time

	Average (in weeks)	Within 1 month (in %)	Within 3 months (in %)	Within 6 months (in %)	Correlation with impact factor		
					Pearson	Sign.	*n*
All	13	19	68	90	−0.29	0.000	2520
Accepted	12						
Rejected	16						
General	11	11	77	96	−0.51	0.000	160
Natural sciences	11	25	77	94	−0.26	0.000	1320
Engineering	13	21	71	89	−0.17	0.000	477
Mathematics and Computer sciences	17	11	54	82	−0.27	0.000	345
Medicine	8	28	84	98	−0.24	0.000	578
Public health	9	27	81	97	−0.25	0.000	318
Psychology	14	11	60	90	−0.21	0.000	313
Economics and Business	18	10	55	82	−0.20	0.001	255
Social sciences	17	8	50	86	−0.10	0.027	452
Humanities	16	7	53	87	−0.07	0.437	130

While writing a peer review may take between 4 and 8 h, in only 19% of all reported cases authors were informed about the outcome in less than a month. In about one third of the cases (32%) authors had to wait 3 months or more and in 10% of the cases even more than 6 months before being informed. Duration differs widely between scientific fields. In Social sciences and Humanities, only 7–8% of the authors were informed within 1 month versus 25% in Natural sciences and 27–28% in Medicine and Public health. In Economics and Business and Mathematics and Computer sciences over one sixth (18%) of authors had to wait 6 months or longer.

It is yet unclear to what extent the long duration of the first review round is the result of the peer review process as such and to what extent it is due to (in)efficient manuscript handling at editorial offices. Given that immediate rejection times are often long (see Table 3 and its discussion below), it seems that inefficiencies at editorial offices also play an important role. The finding that in Medicine and Public Health—where professionalization of journals is relatively high—first response times are the shortest, also points in this direction.

To test this idea further, we looked at the relationship between the journal’s impact factor and first response time. As highly ranked journals generally have more resources at their disposal and thus probably better organized editorial offices, and as reviewers are more motivated to review for those journals, we expected to find a negative relationship. Pearson correlations between first response time and impact factor indeed confirm this expectation. These correlations are significantly negative for all scientific fields combined (*P* = −0.29) as well as for all scientific fields separately, with General journals (*P* = −0.51), Mathematics and Computer sciences (*P* = −0.27), and Natural sciences (*P* = −0.26) having the highest correlations. The only exception was Humanities, where no significant correlation between first response time and impact factor was found. This might be because this field traditionally values publishing books more than publishing in journals (Ware and Mabe 2015).

### Total review duration

Total review duration refers to the time a manuscript is under responsibility of the journal. Besides by the duration of the first review round, total review duration is also determined by the number and duration of subsequent review rounds. Total review duration does not include the time taken by authors to revise and resubmit their manuscript. Given that rejected manuscripts have on average less review rounds, we restrict this analysis to accepted papers.

Table 2 shows that the reported total review duration of accepted manuscripts is on average 17 weeks. Again there are substantial differences between scientific fields. With 12–14 weeks, average total review duration is shortest in Medicine, Public health, and the Natural sciences. It is longest in Economics and Business, where the process takes on average 25 weeks and is twice as long. In Mathematics and Computer sciences, Social sciences and Humanities, total review duration is also long, i.e., 22–23 weeks. Hence the differences in the duration of the review processes we observed for the first review round are also present in the other aspects of the process.

**Table 2:** Total review duration of accepted papers

	Average (in weeks)	Within 1 month (in %)	Within 3 months (in %)	Within 6 months (in %)	Review rounds
All scientific fields	17	11	53	81	2.03
General	17	7	54	83	2.18
Natural sciences	14	13	63	87	1.98
Engineering	17	11	54	81	1.98
Mathematics and Computer sciences	22	4	36	72	2.03
Medicine	12	16	71	92	2.05
Public health	13	14	67	91	1.96
Psychology	20	7	46	76	2.23
Economics and Business	25	7	37	67	2.16
Social sciences	23	3	33	68	2.15
Humanities	22	4	36	71	2.02

If we split out the data further, we note that in Natural sciences, Medicine, and Public health 13–16% of the manuscripts pass through the entire peer review process within 1 month, that this applies to about two thirds of the manuscripts after 3 months, and to 87–92% of the manuscripts after 6 months. In Mathematics and Computer sciences, Social sciences, and Humanities, these figures are 3–4%, one third and slightly above two thirds, respectively. Whereas only 8% of the authors in Medicine had to wait more than 6 months, this applies to one third of authors in Social sciences and Economics and Business.

The total time a manuscript is with the journal is determined by the time a journal takes for a review round and by the number of review rounds. As mentioned in the Background-section, there are indications that the number of review rounds has increased in recent years. In our data, the number of review rounds on average amounts to 2.03, with Psychology (2.23), General journals (2.18), Economics and Business (2.16), and Social sciences (2.15) showing a higher average number of review rounds.

Total review duration correlates significantly and negatively (−0.27) with a journal’s impact factor, thus indicating that total review duration is shorter for higher impact factor journals.

### Immediate (desk) rejection time

Immediate rejection time is the time an editor takes to inform authors that he or she is not interested in the manuscript (and will therefore not send it to reviewers). Our figures clearly show that immediate rejection time is a major source of unnecessary time loss in the peer review process (Table 3). On average, an immediate rejection in Medicine takes 10 days, closely followed by Natural sciences, Public health, and Engineering, taking 11–12 days. Journals in Psychology, Social sciences and Mathematics and Computer sciences take half as long, i.e., 15–17 days. These are relatively high averages, given that in many cases an inspection of the abstract is sufficient to decide that a paper does not fit.

**Table 3:** Immediate rejection time

	Average (in days)	Within 1 week (in %)	Within 2 weeks (in %)	Within 4 weeks (in %)
All scientific fields	12	50	63	83
General	14	46	57	89
Natural sciences	11	54	72	90
Engineering	12	50	63	85
Mathematics and Computer sciences	17	46	54	68
Medicine	10	62	70	92
Public health	12	54	65	79
Psychology	15	32	45	77
Economics and Business	13	47	59	78
Social sciences	15	40	56	71
Humanities	14	50	63	81

On the positive side, in half (50%) of the reported immediate rejection cases, the editor informed the author(s) within 1 week. However, the data also show that in 17% of cases authors had to wait more than 4 weeks to be informed of the rejection. Several authors even had to wait for more than 3 months, or withdrew their manuscripts after hearing nothing for an even longer period. These are clearly unacceptable practices.

The situation is best in Medicine, where 62% of authors are informed about an immediate rejection within 7 days, followed by Natural sciences and Public health where this figure is 54%. Immediate rejection time is longest for authors in the Social sciences and Mathematics and Computer sciences, where in about 30% of reported cases it took the editor 4 weeks or more to inform author(s) that he or she was not interested in the manuscript and would not to send it to reviewers. There is a significant negative correlation (−0.18) between immediate rejection time and the journal’s impact factor, which indicates that journals with a higher impact factor have editors who work faster and editorial offices that are more professionally organized.

Reviewers are generally blamed for long processing times, but our findings indicate that manuscript handling at editorial offices plays an important role too. If editors take a month for an immediate rejection decision, they are probably also slow in finding reviewers and processing referee reports.

### Referee reports

The average number of referee reports is about 2.2 in all scientific fields (see Table 4). This correspondence is remarkable, given the substantial differences between fields in other respects. There is slight variation in the experienced quality of the referee reports between the fields [as indicated on a scale running from 0 (very bad) to 5 (excellent)]. Authors report the quality of the reports to be somewhat higher in Natural sciences, Engineering, and Public health (3.7), and lower in General journals, Psychology, and Economics and Business (3.4). It is interesting that the long review duration in Economics and Business did not translate into referee reports experienced of higher quality.

**Table 4:** Review reports, number, quality and difficulty of requested changes (scale 0–5)

	Review reports	Quality review reports	Difficulty of requested changes	Resulting improvement
All scientific fields	2.2	3.6	2.8	3.7
General	2.2	3.4	3.0	3.7
Natural sciences	2.2	3.7	2.7	3.7
Engineering	2.3	3.7	2.7	3.7
Mathematics and Computer sciences	2.0	3.5	2.6	3.7
Medicine	2.2	3.6	2.8	3.7
Public health	2.2	3.7	2.6	3.7
Psychology	2.2	3.4	3.2	3.7
Economics and Business	2.1	3.4	3.3	3.9
Social sciences	2.3	3.6	3.2	3.8
Humanities	2.1	3.6	2.9	3.9

Authors who were given the opportunity to revise and resubmit their papers were also asked to what extent they perceived the requested changes as difficult and whether they thought their manuscript had improved as a result of the revision. There is a significant positive correlation (0.40) between these factors. When the revision was experienced as more difficult, authors were also more satisfied with the improvement. Regarding the difficulty experienced, revision processes were perceived as easiest in Mathematics and Computer sciences and in Public health (2.6), and as most difficult in Economics and Business (3.3). Regarding the experienced improvement of the manuscript as a result of the revision, authors from Social sciences, Economics and Business, and Humanities reported somewhat higher figures (3.8 and 3.9) compared to the other scientific fields (3.7).

There is a small positive correlation (0.07) between the difficulty experienced regarding the referee reports and the impact factor of the journal. Thus, reviewers of more highly ranked journals tend to make somewhat greater demands on the authors. The degree of improvement experienced regarding the manuscript is not significantly related to impact factor.

### Revision time

The time from the first submission date to the final decision date is not only influenced by the time the manuscript is at the editorial office or being reviewed, but also by the time authors take to revise their manuscript. It is therefore important to look also at the duration of the revision time. Table 5 shows that authors who received a revise-and-resubmit on average take 39 days to revise their manuscript, but there is substantial variation among the fields. Authors in Economics and Business take longest to revise their manuscripts: on average 64 days to prepare and submit a revised version. This is substantially longer than authors in Natural sciences, Engineering and Mathematics and Computer sciences (32–34 days) and in Public health (29 days). Apparently, in Economics and Business it is not only the editors who take more time.

**Table 5:** Revision time

	Average (in days)	Within 1 week (in %)	Within 1 month (in %)	Within 3 months (in %)
All scientific fields	39	15	61	92
General	40	14	58	92
Natural sciences	34	17	66	94
Engineering	32	18	71	95
Mathematics and Computer sciences	33	18	69	95
Medicine	38	15	60	93
Public health	29	18	71	99
Psychology	50	12	46	90
Economics and Business	64	3	39	79
Social sciences	50	9	49	87
Humanities	47	10	52	90

Table 5 also shows the percentage of manuscripts revised within a specific number of days. While 18% of authors in Engineering, Mathematics and Computer sciences and Public health revise their manuscript within 7 days, this applies to 9–10% of authors in Social sciences and Humanities and only 3% of authors in Economics and Business.

Regarding the relationship between the journal’s impact factor and the time authors take to revise their manuscript, we expected authors who received a revise-and-resubmit from a high-level journal to be more motivated to complete the revision of their manuscript quickly. However, no significant correlation was found between revision time and the journal’s impact factor.

### Rating of peer review experience

The SciRev questionnaire gives authors the opportunity to provide an overall rating of the review experience on a scale from 0 (very bad) to 5 (excellent); see Table 6 for details. Authors of accepted manuscripts give the peer review process a much higher rating (4) than authors of rejected manuscripts (2.2). Moreover, the rating of the peer review process is negatively related to total review duration. This correlation is −0.43 for both accepted and rejected manuscripts.

**Table 6:** Rating of review process of accepted and rejected papers per field (scale 0–5)

	Accepted papers	Rejected papers
All scientific fields	4.0	2.2
General	3.8	1.3
Natural sciences	4.0	2.2
Engineering	4.0	2.4
Mathematics and Computer sciences	4.0	1.8
Medicine	4.0	2.0
Public health	4.1	1.7
Psychology	3.9	2.4
Economics and Business	4.1	2.4
Social sciences	4.0	2.5
Humanities	4.0	2.3

To determine how the various factors might affect the satisfaction of authors with the peer review process, we turn to the results of multivariate analyses (see Table 7). The first columns show the results of Model 1, which contains all relevant variables. Model 2 contains the same variables but also the significant interactions between the variables.

**Table 7:** Regression analysis with overall rating as dependent variable (scale 0–5)

	Model 1			Model 2		
	*B*	*t*	Sign.	*B*	*t*	Sign.
Paper is accepted	2.03	37.07	0.00	1.59	8.34	0.00
First response time	−0.04	−22.58	0.00	−0.05	−8.19	0.00
Number of review rounds	−0.40	−12.18	0.00	−0.51	−4.33	0.00
Number of review reports	0.12	5.51	0.00	0.22	4.17	0.00
Log of impact factor	0.05	1.04	0.30	0.11	1.20	0.23
English is first language	−0.14	−3.12	0.00	−0.37	−2.42	0.02
Scientific field^a^						
General	−0.44	−4.52	0.00	−0.39	−4.08	0.00
Natural sciences	−0.12	−2.93	0.00	−0.10	−2.53	0.01
Engineering	0.04	0.53	0.60	0.04	0.58	0.56
Mathematics and Computer sciences	0.12	1.72	0.08	0.12	1.75	0.08
Medicine	−0.22	−3.66	0.00	−0.22	−3.77	0.00
Public health	−0.23	−2.57	0.01	−0.22	−2.51	0.01
Psychology	0.18	2.54	0.01	0.16	2.35	0.02
Economics and Business	0.33	4.11	0.00	0.30	3.84	0.00
Social sciences	0.27	4.26	0.00	0.22	3.51	0.00
Humanities	0.07	0.57	0.57	0.09	0.70	0.48
Interactions						
Paper is accepted*						
-First response time				0.01	2.80	0.01
-Number of review rounds				0.40	4.55	0.00
-Number of review reports				−0.27	−5.37	0.00
-Log impact factor				0.19	2.18	0.03
-English is first language				0.27	2.71	0.01
First response time*						
-Number of review rounds				−0.01	−2.25	0.02
-Number of review reports				0.01	3.17	0.00
-English is first language				−0.01	−3.66	0.00
Number of review rounds*						
-Log impact factor				−0.12	−2.32	0.02
Number of review reports*						
-English is first language				0.11	2.08	0.04

^a^ For field dummies deviation from mean (effects) coding is used

As can be seen in Model 1, all variables, except impact factor, are significantly related to authors’ rating of the peer review process of their manuscript. As expected, authors of accepted manuscripts rate the process significantly more positive than authors of rejected manuscripts. Authors tend to suffer from attributional bias: if their paper is rejected, they often blame this on situational factors such as incompetent reviewers and uninterested editors; but if it is accepted they tend to attribute this to their own expertise and competence in writing high-quality papers (Garcia et al. 2016).

Authors also value speed of the peer review process. When the duration of the first review round is shorter and there are fewer review rounds, authors give the process a significantly higher rating. Authors who receive more referee reports also tend to be more positive about the process. Their perception might be that their manuscript has been dealt with more seriously and thoroughly. Authors from countries where English is the first language rate the peer review process less positive than authors from other countries. It is possible that these authors have higher expectations of the process and are more critical regarding aspects that do not meet their expectations.

Taking into account other factors, authors in Economics and Business, Social sciences, Psychology, and Mathematics and Computer sciences are more positive about the peer review process than authors in Natural sciences, Medicine, Public health, and especially General journals.

When we include the significant interactions in the model (Model 2), the sign and significance of the main effects stay the same. The interaction analysis shows that the negative effect of a longer duration of the first review round and the negative effect of more review rounds are less profound for accepted papers. Hence it seems that authors are willing to accept extensive revision work if this is rewarded with the acceptance of their paper. At the same time, they seem especially disappointed if the manuscript is still rejected after a long review process.

The negative interaction between a paper being accepted and the number of referee reports indicates that authors of rejected papers may consider a higher number of reports as a sign that their paper was taken seriously and might be content with extensive feedback. For obvious reasons, authors of accepted papers are more positive when the journal has a higher impact factor. Authors from English-speaking countries are less negative about the peer review process when their paper is accepted and when they receive more referee reports but find a long process more problematic. This might reflect that they have higher expectations that their paper will be accepted and that the peer review process will be short and efficient compared to authors from non-English-speaking countries.

When the duration of the first review round is longer, or when the impact factor of the journal is higher, authors are more concerned about a higher number of review rounds. In those cases, they might expect a smooth continuation of the process and be more disappointed when this proves not to be the case. A longer duration of the first review round is considered less negative by authors who receive more referee reports.

## Qualitative findings

The motivations authors give for their rating of the peer review process on SciRev.sc contain important qualitative information on author experiences. We analyzed these motivations and registered the author’s major concern(s). A first important observation is that about half (918) of the 1879 comments is positive. Many authors, in particular of accepted papers, are satisfied with the process and express their gratitude in their motivations. Of the 961 comments with a negative connotation, 371 (39%) express concerns about the duration of the review process. This aspect of long review duration is included in the quantitative outcomes and has been discussed in the preceding sections.

A more informative source of discontent, mentioned 437 times (45%), concerns the role of editors and editorial offices. Poor communication of editors/offices—in particular not reacting to information requests—are a major source of frustration mentioned by authors. We received reports of authors who waited over 6 months without hearing anything of the journal or receiving reactions to information requests. Also editors who ‘hide’ behind reviewers and do not take an independent position vis-à-vis them are perceived as problematic. In particular when referee reports are contradictory—as often happens—it is important that editors provide guidance and indicate the comments on which authors should focus in their revision.

Poor quality of referee reports is mentioned in 141 (15%) of the critical comments. Referee reports are often perceived to be superficial, contradictory, unreadable, ask unreasonable modifications, or convey the impression that the reviewer did not read or understand the paper. Some other issues mentioned are the addition of completely new comments in the second review round, the theft of ideas, or asking for unnecessary references.

## Conclusion

In this paper we study various aspects of the peer review process on the basis of 3500 review experiences reported in the last 3 years on the SciRev.sc website. Aspects discussed include the first response time (duration of the first review round), total review duration (the time the manuscript is at the editorial office or with reviewers), the immediate rejection time, the time authors take for their first revision (revision time), the number, quality, and difficulty of referee reports received, and the overall rating of the process.

We find considerable variation between the ten scientific fields distinguished. Whereas the reported first response time is 8–9 weeks for Medicine and Public health, it is 11–14 weeks in Natural sciences, Engineering, Psychology, and General journals and 16–18 weeks in Economics and Business, Social sciences, Mathematics and Computer sciences, and Humanities (Table 1). There is also considerable variation around these averages. While 27–28% of authors in Medicine and Public health were informed within a month, 18% of authors in Mathematics and Computer sciences and Economics and Business had to wait more than 6 months for a decision. As expected, these figures also translate into longer total review durations reported for the scientific fields with longer first review rounds (Table 2).

The long duration of the peer review process is often blamed on reviewers taking much time to complete their reports. However, our figures indicate that inefficient editorial processes are also important. The reported immediate rejection time (Table 3), which is not influenced by reviewers, shows substantial variation among the fields and is often unreasonably long. Whereas in half of the immediate rejection cases authors were informed within a week, in about one sixth of these cases authors had to wait for more than 4 weeks. Medicine performs best with an average of 10 days, Natural sciences, Public health and Engineering come second with 11–12 days. Psychology, Social sciences, and Mathematics and Computer sciences take longest with 15–17 days. If editors take much time for a desk rejection, it is likely they also take much time finding reviewers and processing incoming referee reports. Immediate rejection time is therefore a powerful indicator of the overall performance of editorial offices.

The total time between submission of a manuscript and the final decision of the editor is not only influenced by the time reviewers take to submit their reports and the time editorial offices take to handle the manuscript, but also by the time authors take to revise and resubmit their manuscript (Table 5). In this respect, the situation is similar to that of the other durations. While, on average, authors take 39 days to revise their manuscript, authors in Psychology and Social sciences take 50 days, and those in Economics and Business even 64 days. On the other hand, authors in Public Health, Engineering, Mathematics and Computer sciences, and Natural sciences take only 29–34 days for a revision. The longer duration in some fields is not associated with a higher number of referee reports (2.0–2.3) nor with more difficult referee reports (2.6–3.3).

Most characteristics of the peer review process studied are related to the journal’s impact factor. More highly ranked journals have a shorter duration of the first review round (*P* = −0.29), total review duration (*P* = −0.27), and immediate rejection time (*P* = −0.18), all indicating that review processes of more highly ranked journals are more efficient. We also found a small but significant positive correlation (*P* = 0.08) between experienced difficulty of the referee reports and impact factor, indicating that reviewers of more highly ranked journals are somewhat more demanding.

As expected, authors of accepted manuscripts are more satisfied with the peer review experience than authors of rejected papers (Table 6). On a scale from 0 (very bad) to 5 (excellent), they rate the process a 4, compared to a 2.2 for authors of rejected manuscripts. A longer duration of the process is negatively associated with the rating, independent of the process outcome. For both accepted and rejected manuscripts the Pearson correlation coefficient between total review duration and rating is −0.37.

To assess the independent associations between the characteristics of the process and the satisfaction of authors, a multivariate regression analysis was performed with the overall rating of the process as dependent variable (Table 7). This analysis shows that even when the other variables are taken into account, all three aspects, i.e., a shorter duration of the first review round, a lower number of review rounds, and acceptance of the paper, are associated with a significantly higher overall rating of the experience. Interestingly, it also shows that, in spite of the longer duration in Economics and Business, Social sciences, and Mathematics and Computer sciences, authors in those fields are more positive about the process than authors in the General journals, Medicine and Public health, where processes are shorter. Expectations thus clearly play a role.

As expected, authors of accepted papers are even more positive if the journal has a higher impact factor. They are (afterwards) also less bothered by a longer duration of the first review round and by more than one review round. We also find that authors rate the process more positive if they receive more referee reports, in particular after a long first review round and when the manuscript is rejected. This indicates that authors appreciate the work of reviewers and the feedback given on their manuscripts. Compared to authors from non-English-speaking countries, those from English-speaking countries are generally less satisfied with the process, particularly when their manuscript is rejected or in case of more than one review round. This suggests that authors from English-speaking countries have higher expectations of the peer review process.
